# Supplementary material for: Adaptation and selection shape clonal evolution of tumors during residual disease and recurrence
Source: Nat Commun. 2020 Oct 6;11:5017. doi: 10.1038/s41467-020-18730-z (PMC7539014; doi:10.1038/s41467-020-18730-z)
Supplement: Supplementary file 1 — Supplementary Information [file 41467_2020_18730_MOESM1_ESM.pdf]

Supplementary Information

Walens et al., Adaptation and Selection Shape Clonal Evolution of Tumours During Residual Disease and Recurrence

Supplementary Figures 1-10
Supplementary Tables 1-4

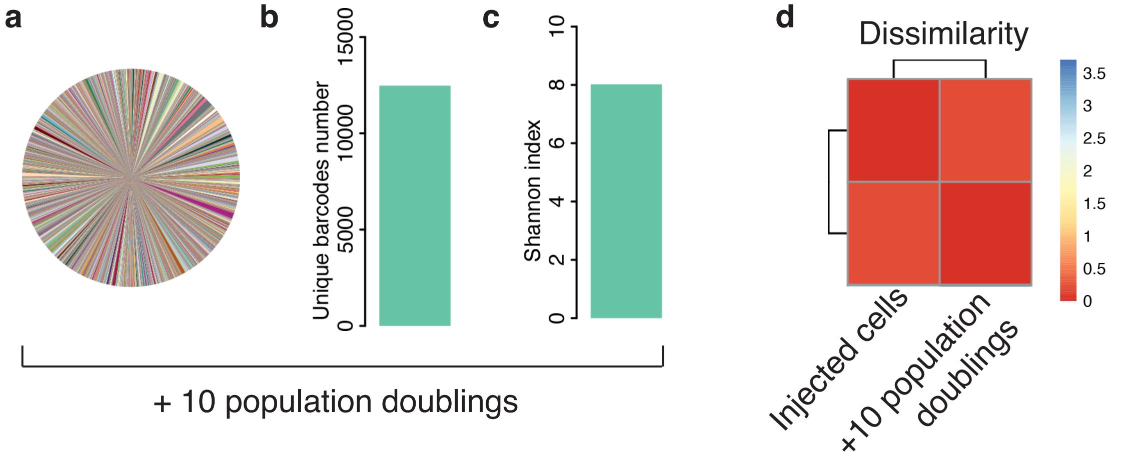

Supplementary Figure 1. Stability of barcoded cell population. a. Pie chart showing barcode abundance of the injected cell population following passaging for an additional 10 population doublings. **b.** The number of unique barcodes detected in cells following 10 additional population doublings. n=1 sample sequenced following 10 population doublings. **c.** The Shannon Diversity Index of cells following 10 additional population doublings. n=1 cell population following 10 doublings. **d.** The Jensen-Shannon Divergence between the injected cell population and cells following 10 additional population doublings.

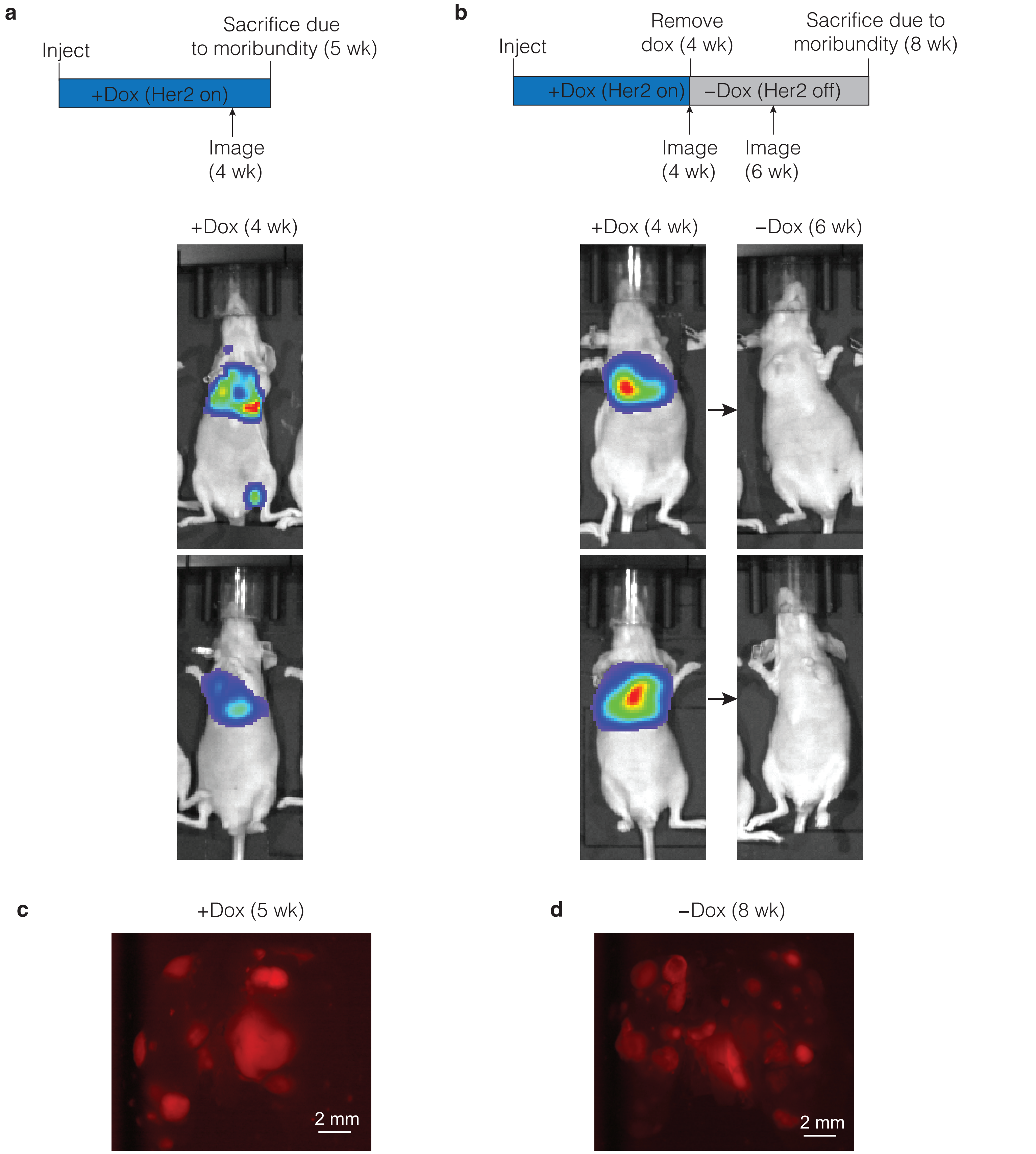

Supplementary Figure 2. Response of lung metastases to Her2 downregulation. a. - b. Barcoded primary tumor cells were injected into the tail vein of recipient mice on dox. Four weeks later mice luciferase imaging was performed to detect lung metastasis. One cohort of mice was sacrificed due to moribundity 5 weeks following injection (a). A second cohort of mice had dox removed at 4 weeks to induce Her2 downregulation (b). Imaging of this cohort at 6 weeks confirmed loss of luciferase signaling, which is a surrogate for Her2 expression. This cohort of mice was sacrificed at 8 weeks due to moribundity. **c. - d.** Lungs from mice with Her2 on (+Dox, c) or Her2 off (-Dox, d) were imaged with a fluorescent dissecting microscope to visualize lung metastases. Imaging was performed on 4 independent tumors from each cohort. Representative images of tumors with Her2 on (+Dox) or Her2 off (-Dox) are shown. Scale bar = 2 mm.

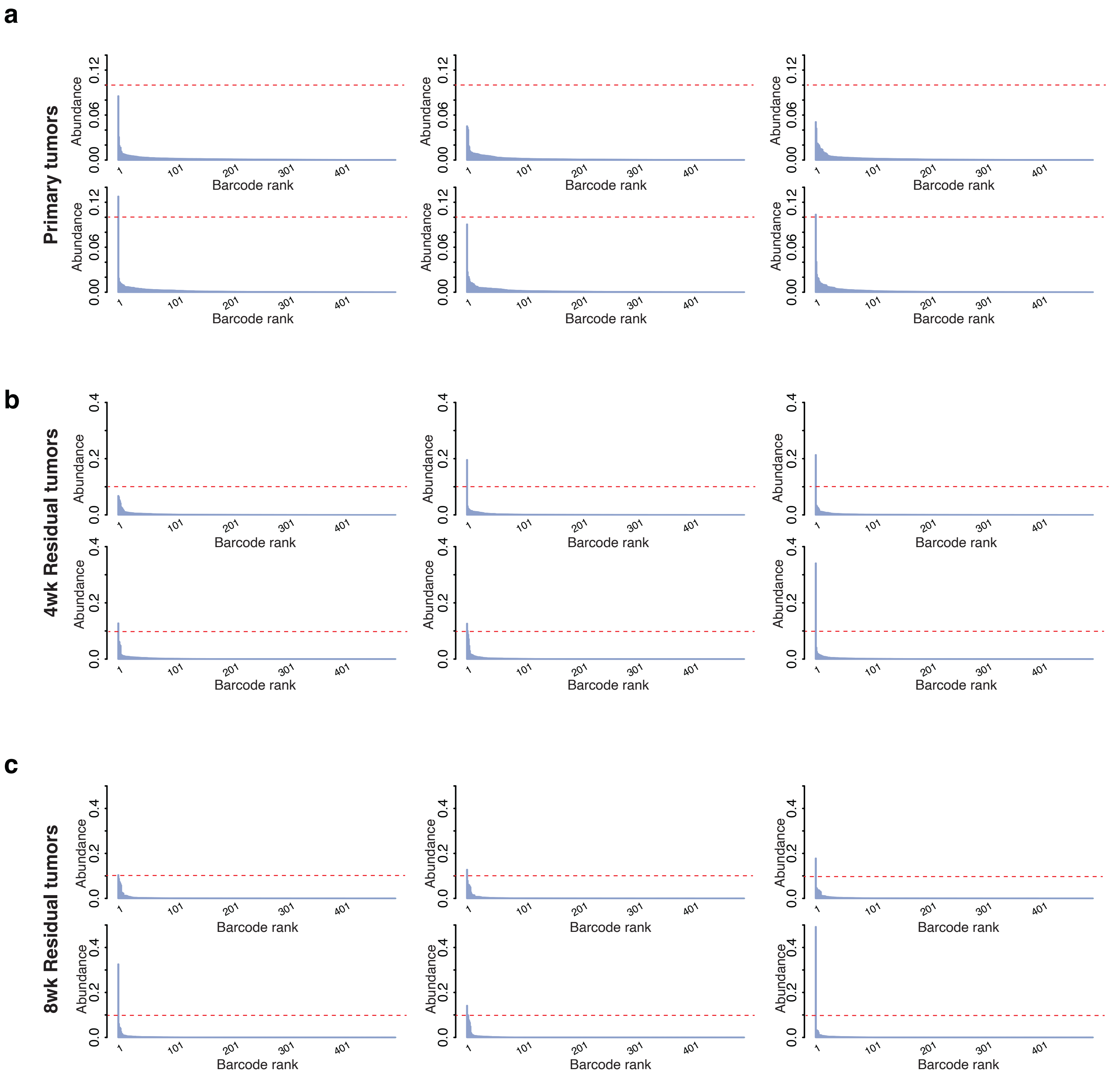

Supplementary Figure 3. Barcode distribution in primary and residual tumors. a. Barcode abundance in primary tumors. **b.** Barcode abundance in early residual tumors (4 weeks following dox withdrawal). **c.** Barcode abundance in late residual tumors (8 weeks following dox withdrawal). For all plots, barcodes are ranked on the x-axis from most to least abundant. Red line denotes 10% abundance.

a

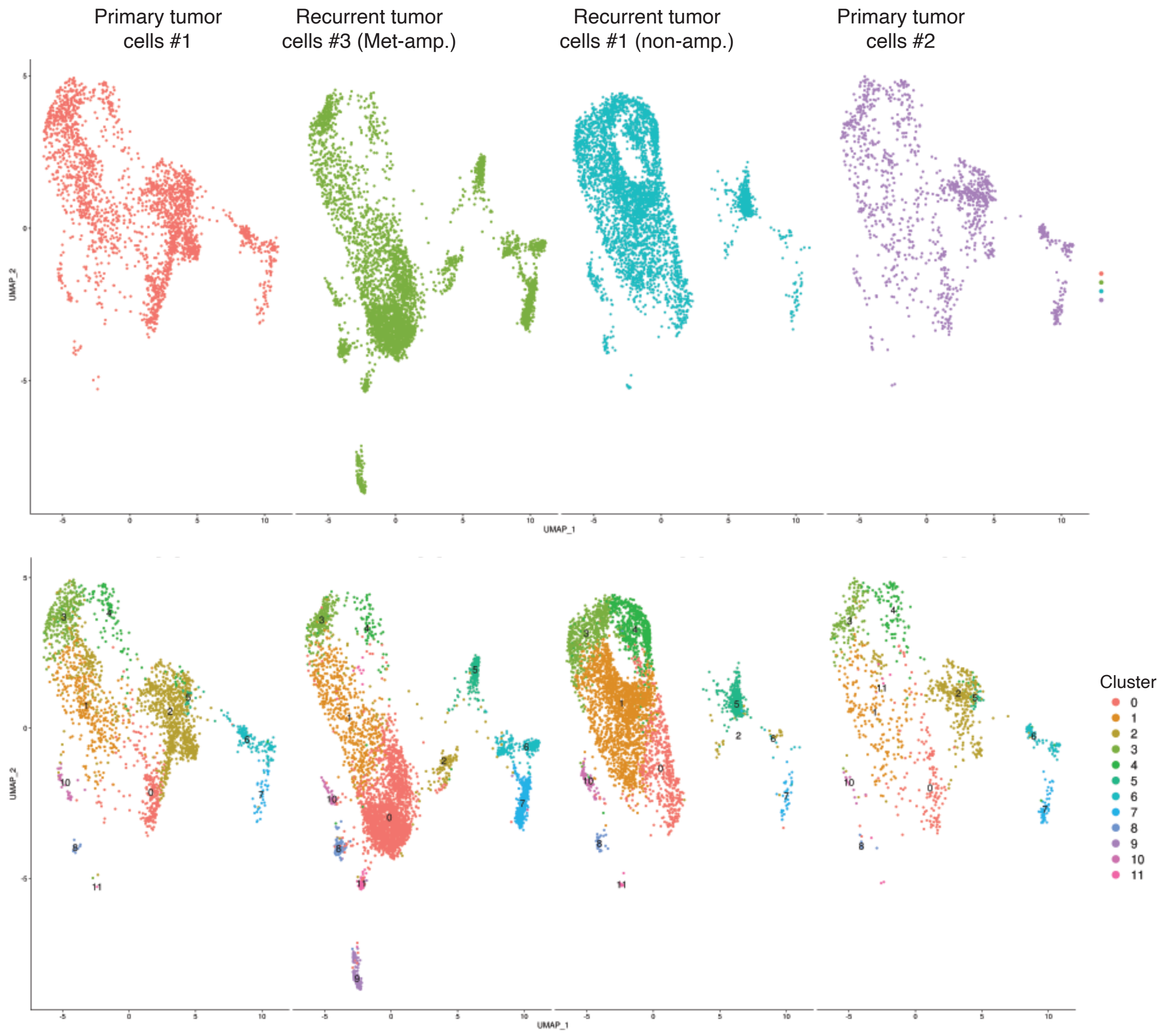
Primary tumor cells #1
Recurrent tumor cells #3 (Met-amp.)
Recurrent tumor cells #1 (non-amp.)
Primary tumor cells #2
UMAP_1
UMAP_2
Cluster
0
1
2
3
4
5
6
7
8
9
10
11

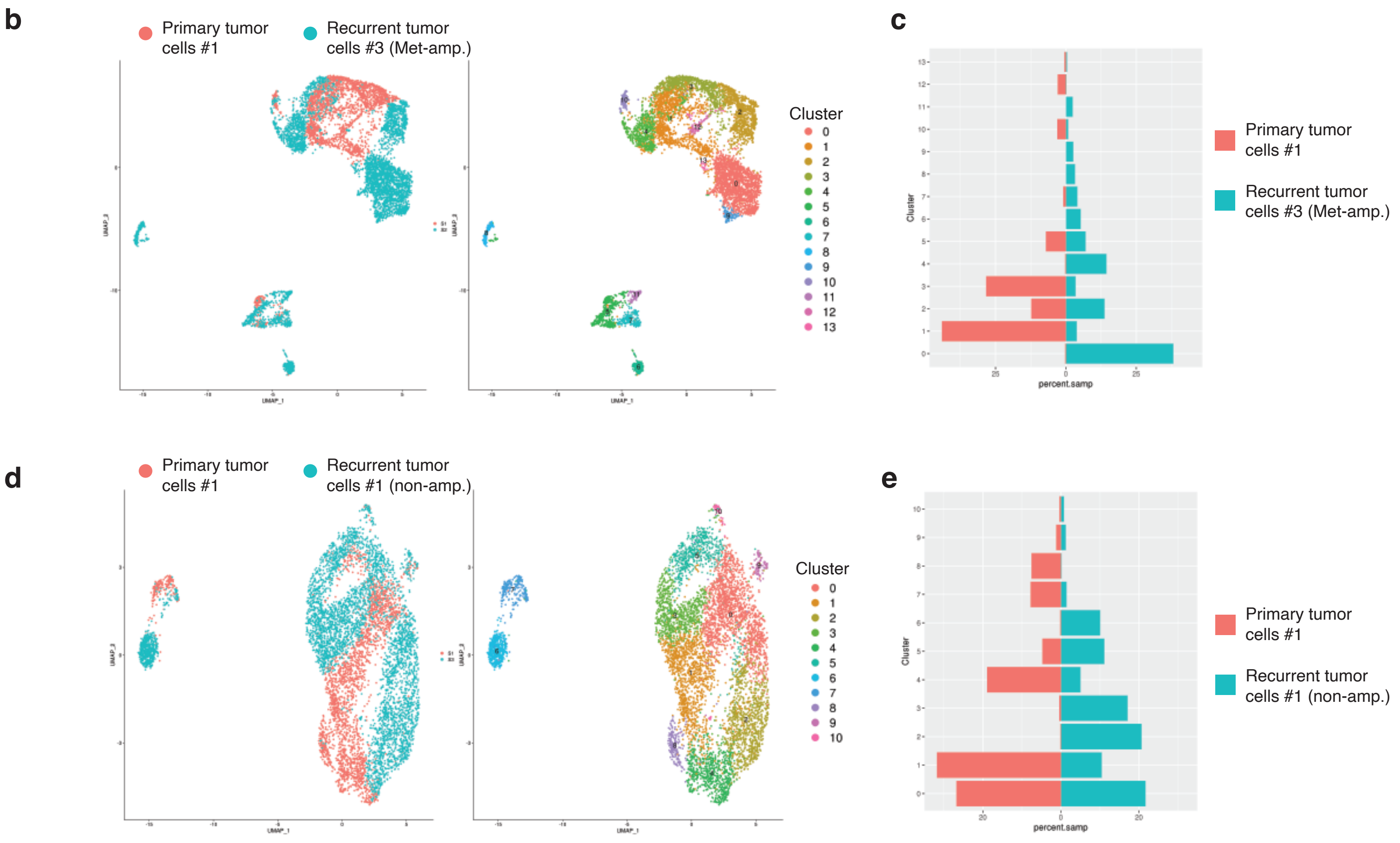

Supplementary Figure 4. scRNA-seq of primary and recurrent tumor cell lines. a. UMAP plots of scRNA-seq data showing cell clusters in primary and recurrent tumor cell lines. **b.** UMAP plots of pair-wise-comparison between primary tumor cell line #1 and recurrent tumor cell line #3. Cluster ID is shown at right. **c.** Relative abundance of primary and recurrent tumor cells found in each cluster. Cluster #7 was predominantly composed of recurrent tumor cells but had a small number of primary tumor cells. n=1 cell primary cell line and n=1 recurrent cell line. **d.** UMAP plots of pairwise-comparison between primary tumor cell line #1 and recurrent tumor cell line #1. Cluster ID is shown at right. **e.** Relative abundance of primary and recurrent tumor cells found in each cluster. Clusters #2 and 3 were predominantly composed of recurrent tumor cells but had a small number of primary tumor cells. n=1 cell primary cell line and n=1 recurrent cell line.

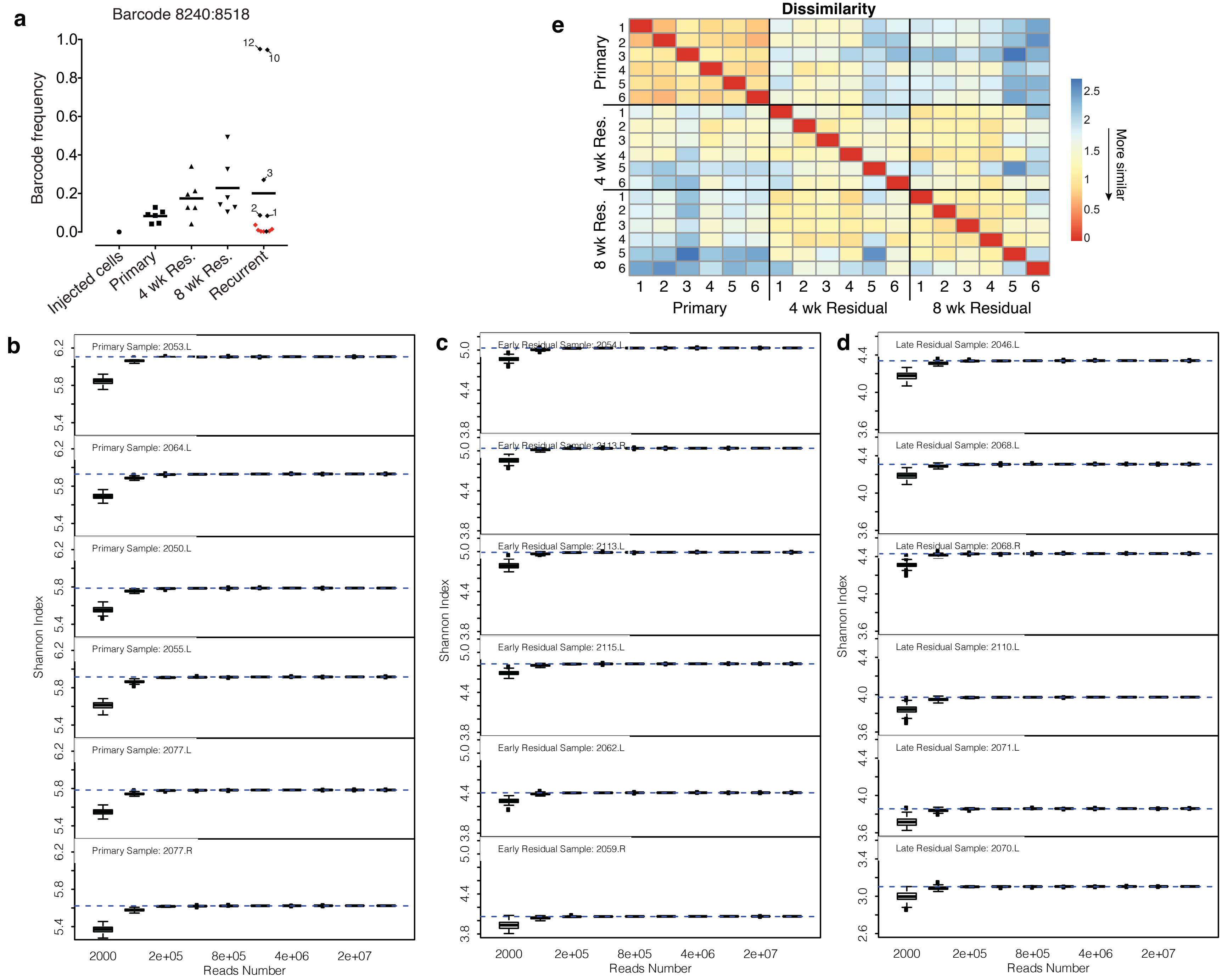

Supplementary Figure 5. Changes in barcode composition during residual disease. a. The frequency of Barcode 8240:8518 in primary, residual, and recurrent tumors. Individual recurrent tumors are labeled, and Met-amplified recurrent tumors are shown in red. n=1 for the injected cell population, n=6 biologically independent primary tumors, n=6 biologically independent 4-wk residual tumors, n=6 biologically independent 8-wk residual tumors, and n=12 biologically independent recurrent tumors. **b.– d.** Simulation experiments showing the Shannon Diversity Index for each tumor following randomly sampling of decreasing numbers of reads from each tumor (range: 2,000 – 2x10^8). The Shannon Index was lower in residual tumors as compared to primary tumors across the entire range of simulated reads. **e.** Correlation matrix showing the similarity in barcode abundance between samples demonstrating that the barcode distribution progressively changes during residual disease. The Jensen-Shannon divergence was used to measure dissimilarity among tumors.

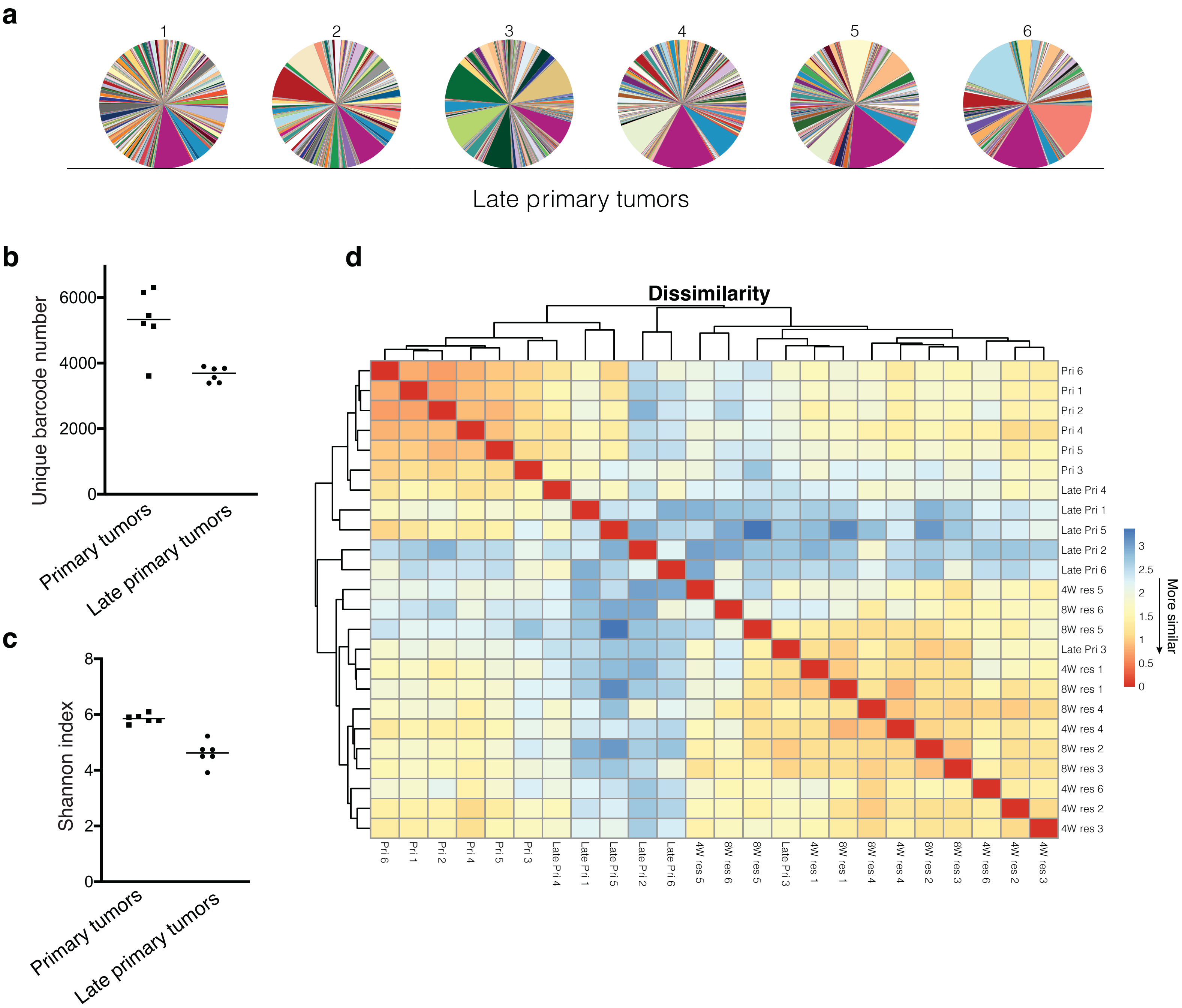

Supplementary Figure 6. Changes in barcode composition during continued tumor growth in the presence of Her2. a. Pie charts showing the relative frequency of barcodes in 6 independent late primary tumors. Note that individual barcodes are represented by the same color in each pie chart. **b.** Number of unique barcodes detected in 6 independent late primary tumors. **c.** Shannon diversity index showing barcode complexity of 6 independent late primary tumors. **d.** Correlation matrix showing the similarity in barcode abundance between samples. Most late primary tumors cluster with primary tumors and are dissimilar from early and late residual tumors. The Jensen-Shannon divergence was used to measure dissimilarity among tumors.

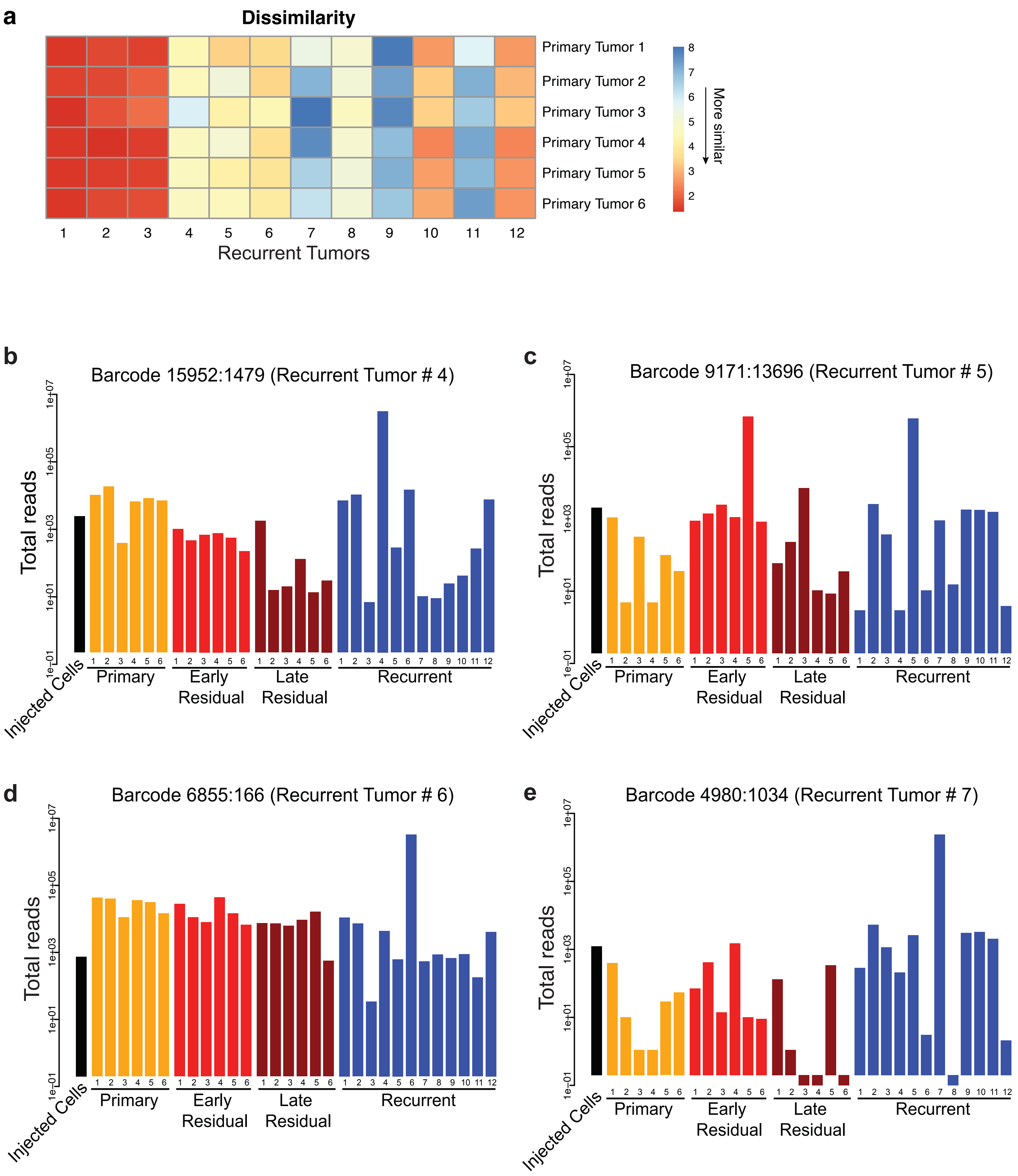
a
Dissimilarity
Primary Tumor 1
Primary Tumor 2
Primary Tumor 3
Primary Tumor 4
Primary Tumor 5
Primary Tumor 6
8
7
6
5
4
3
2
More similar
1 2 3 4 5 6 7 8 9 10 11 12
Recurrent Tumors
b
Barcode 15952:1479 (Recurrent Tumor # 4)
c
Barcode 9171:13696 (Recurrent Tumor # 5)
d
Barcode 6855:166 (Recurrent Tumor # 6)
e
Barcode 4980:1034 (Recurrent Tumor # 7)
Total reads
1e+07
1e+05
1e+03
1e+01
1e-01
Injected Cells
Primary
Early Residual
Late Residual
Recurrent

f

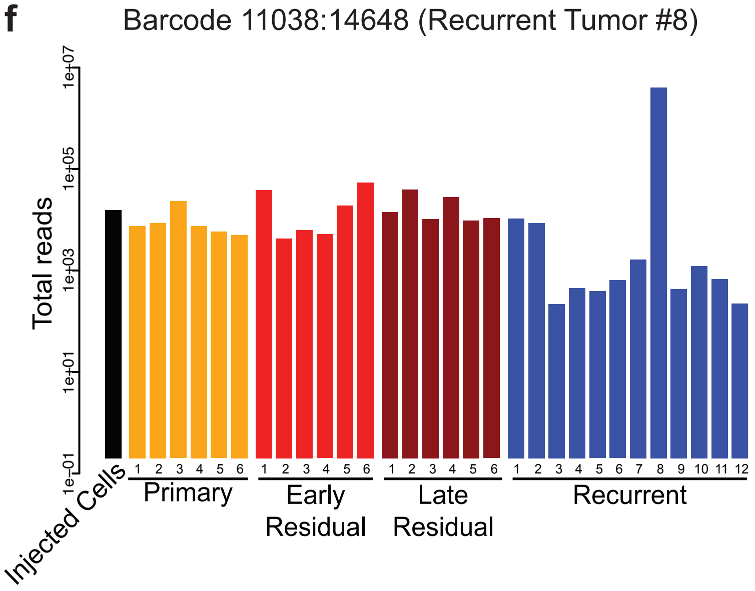

g

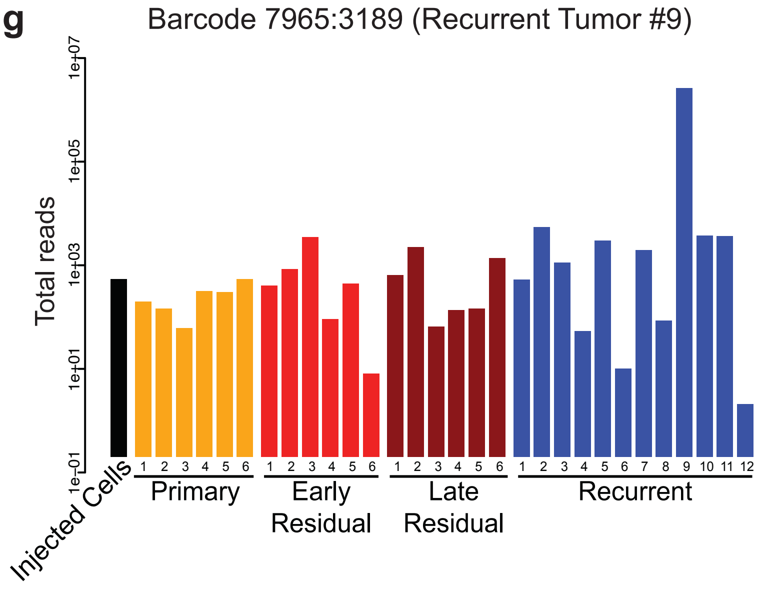

h

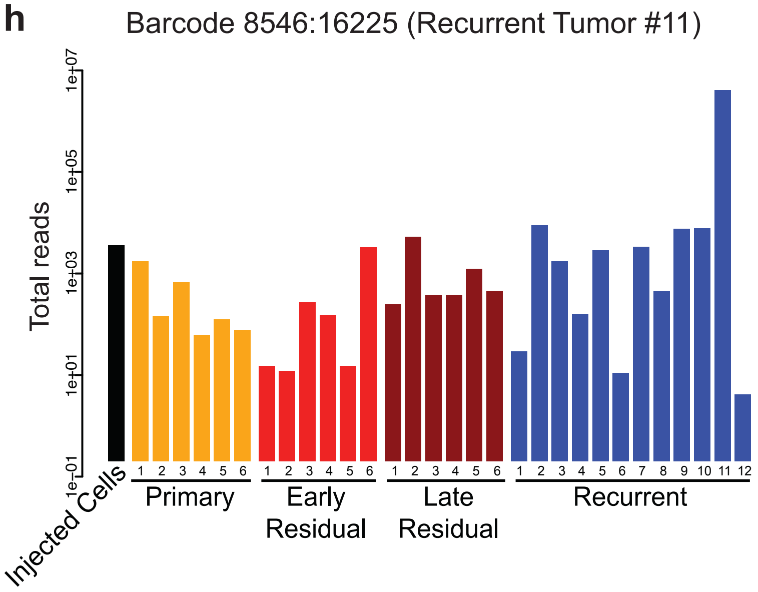

Supplementary Figure 7. Unique barcode composition in individual recurrent tumors. a. Correlation matrix showing the similarity in barcode abundance between recurrent tumors and primary tumors. Recurrent tumors fall into three groups, one group (#1-3) whose barcode distribution is highly similar to primary tumors, one group (#10 and 12) with intermediate similarity, and one group (#4-9, 11) whose barcode distribution is very dissimilar. The Jensen-Shannon divergence was used to measure dissimilarity among tumors. **b.– h.** The number of reads across all tumors for the most abundant barcodes in recurrent tumors #4-9 and 11. For (b) – (h), n=1 for the injected cell population, n=6 biologically independent primary tumors, n=6 biologically independent 4-wk residual tumors, n=6 biologically independent 8-wk residual tumors, and n=12 biologically independent recurrent tumors.

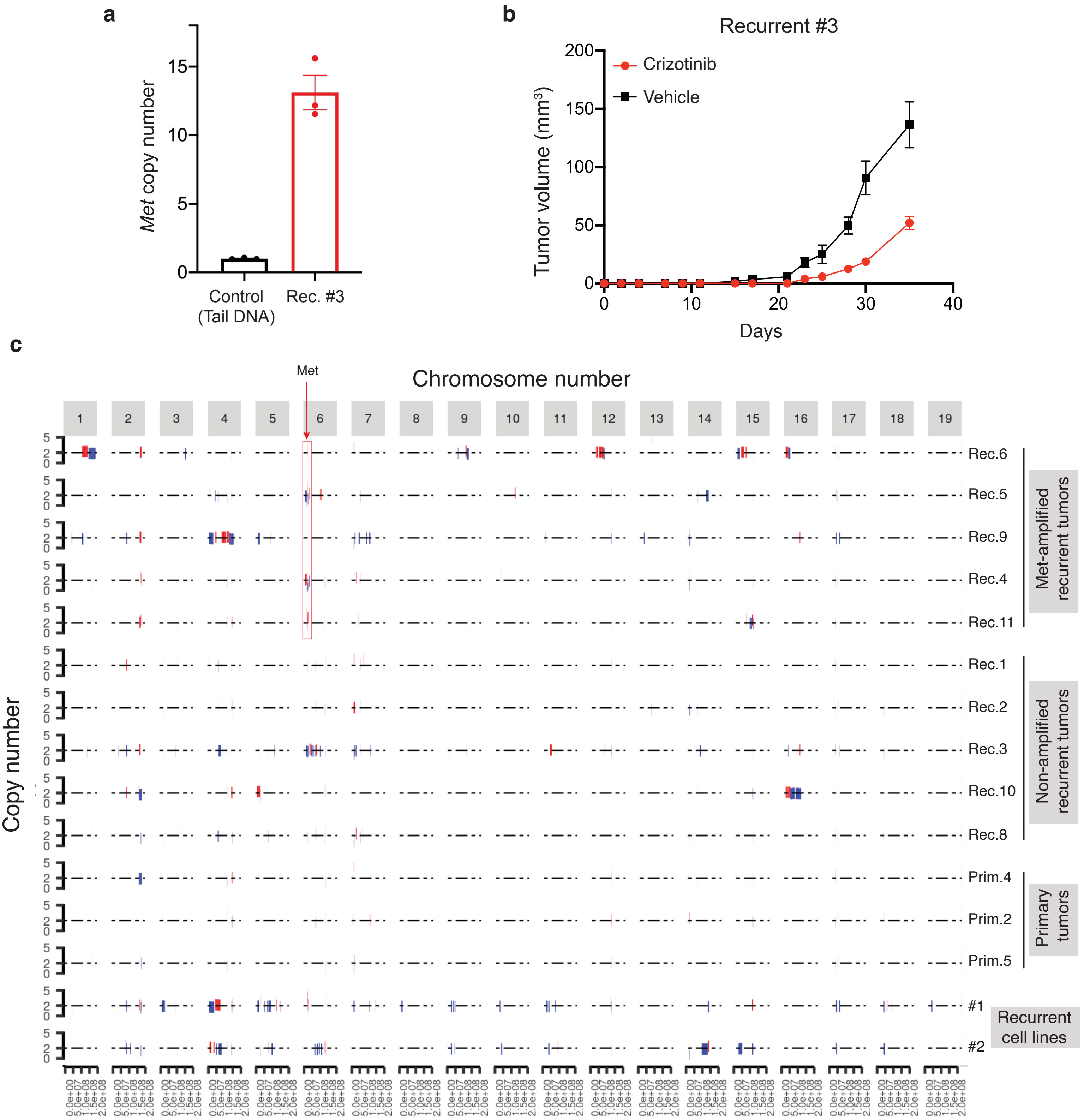

Supplementary Figure 8. Met-dependence and genomic alterations in recurrent tumors. **a.** Met copy number in recurrent tumor #3. n=3 biologically independent tail samples and n=1 recurrent tumor cell line examined over 3 independent experiments. Data are presented as mean ± SEM. **b.** Tumor growth curves for orthotopic tumors formed from recurrent tumor #3 cells and treated with vehicle or the Met inhibitor Crizotinib. n=8 biologically independent orthotopic recurrent tumors per cohort. Data are presented as mean ± SEM. **c.** Copy-number alterations across all 19 chromosomes. Copy-number gains are shown in red and copy-number losses are shown in blue. Tumors are grouped by cohort. The location of Met on chromosome 6 is shown in red.

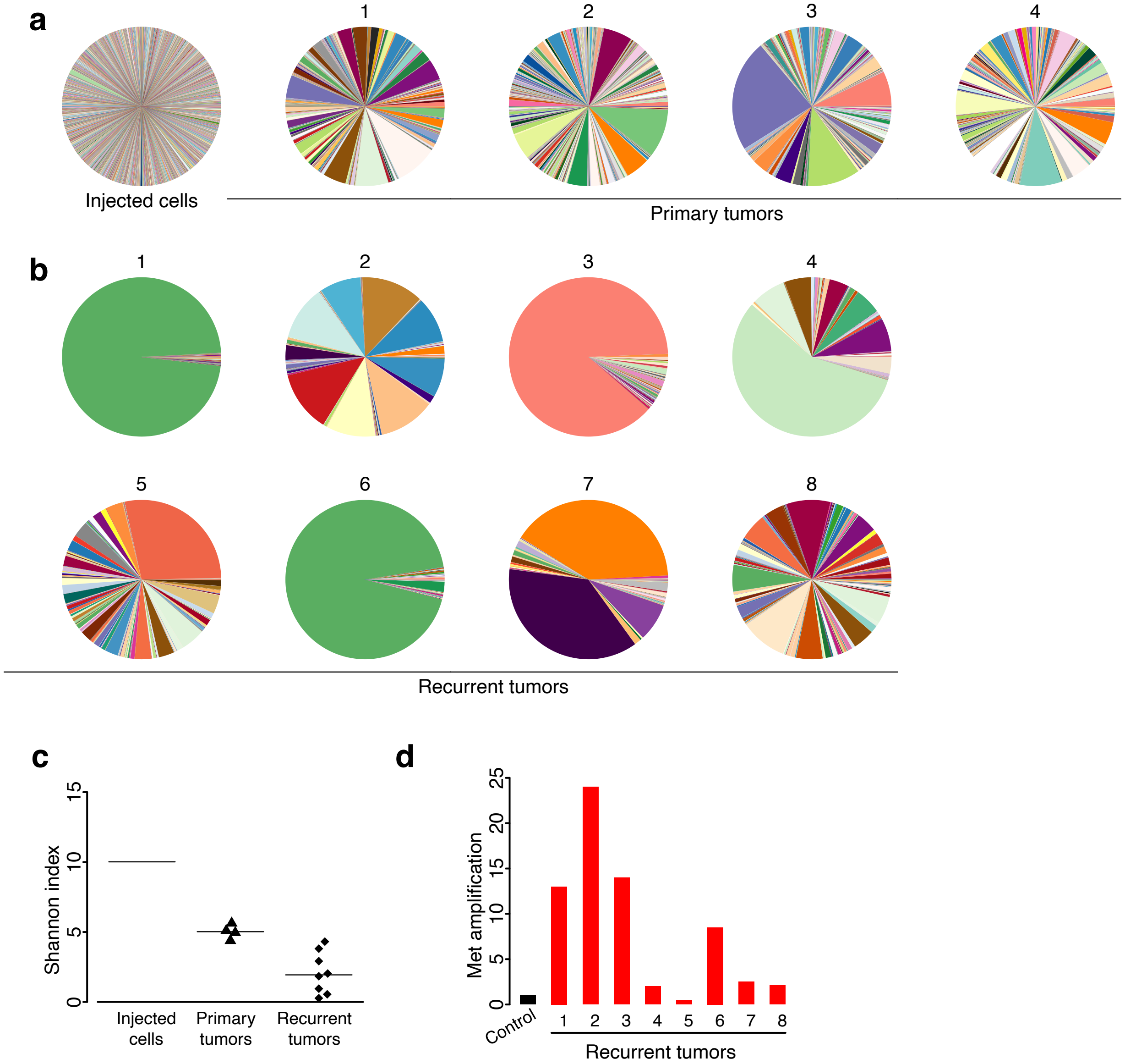

Supplementary Figure 9. Changes in clonal complexity and Met amplification in recurrent tumors arising from an independent donor tumor. a. An independent donor tumor (donor tumor #2) was infected with the barcode library and injected into mice as in Figure 1. Pie charts show barcode abundance in the injected cell population and 4 independent primary tumors. **b.** Pie charts showing the relative frequency of barcodes in 8 independent recurrent tumors. **c.** Shannon diversity index showing barcode complexity of the starting cell population, 4 primary tumors, and 8 recurrent tumors. **d.** qPCR analysis of Met copy number in primary and recurrent tumors. Data are expressed as fold-increase in Met copy number relative to blood. n=3 biologically independent control samples and n=8 biologically independent recurrent tumors.

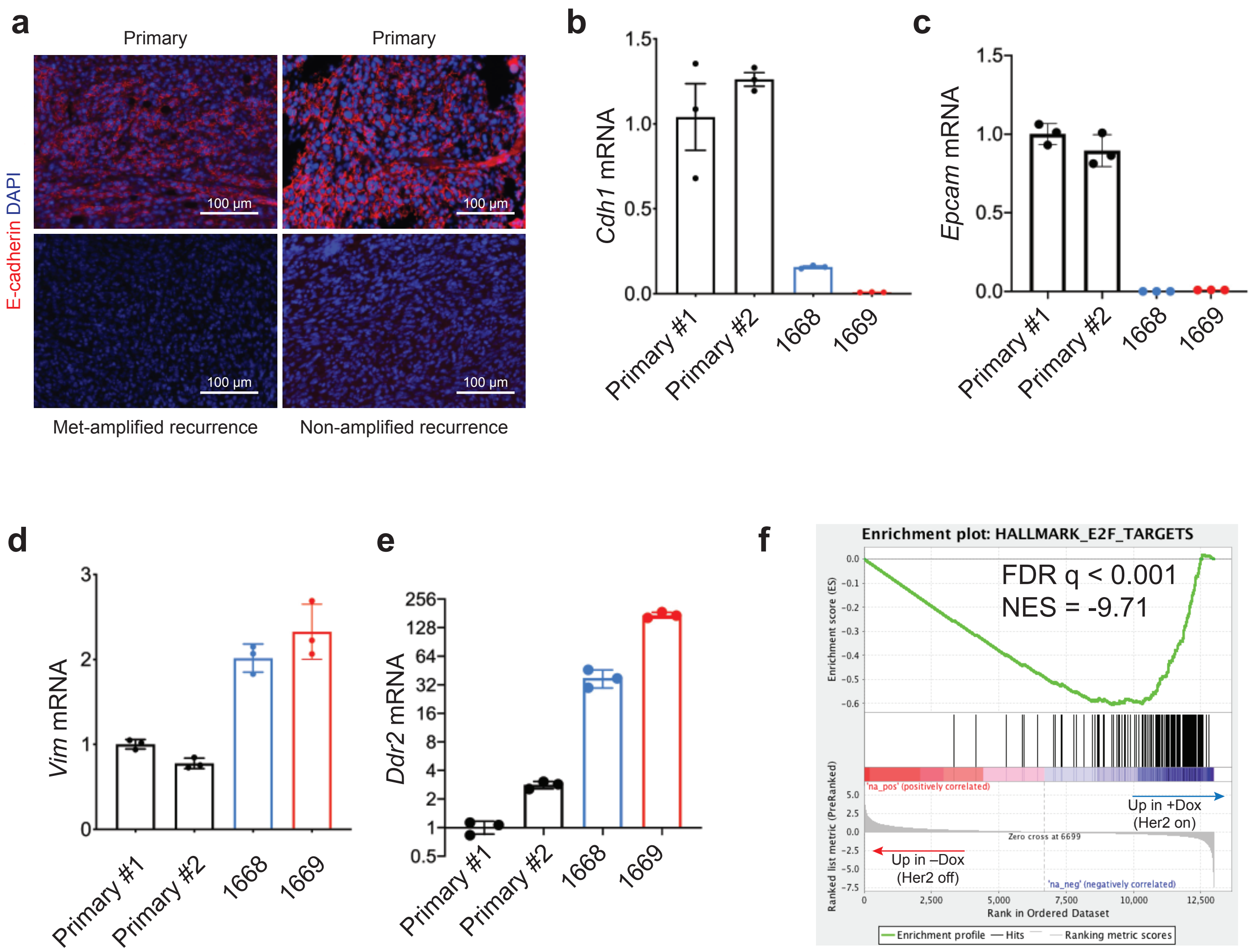

Supplementary Figure 10. Adaptive EMT in recurrent tumors. a. Immunofluorescence staining for E-cadherin in primary or recurrent orthotopic tumors from Figure 5. Staining was performed on 3 independent tumors from each cohort. Representative images of primary tumors and recurrent tumors with and without Met amplification are shown. Scale bar = 100 µm. **b-e.** qRT-PCR analysis showing expression of epithelial (Cdh1 and Epcam) and mesenchymal (Vim and Ddr) markers in cells derived from primary tumors (donor tumor #1 and 2), or from orthotopic recurrent tumors with (1669) or without (1668) Met amplification. For b.– e., n=2 biologically independent primary tumor cell lines, n=1 recurrent cell line without Met amplification, and n=1 recurrent tumor cell line with Met amplification, examined over 3 independent experiments. Data are presented as mean ± SEM. **f.** Gene set enrichment analysis showing enrichment of an E2F signature in cells grown in the presence of Dox (Her2 on). Normalized enrichment score was calculated using the Kolmogorov-Smirnov statistic. To correct for multiple testing, the FDR q-value was estimated using permutation testing to compare the actual NES to random gene sets.

Supplementary Table 1. Amino acid changes resulting from non-synonymous single-nucleotide variants in the 20 most commonly mutated genes in human breast cancer from cBioPortal. The known activating mutation in PIK3CA is shown in red.

Gene name	Recurrent tumor cell lines		Non-Met Amplified Recurrent Tumors					Primary Tumors			Met Amplified Recurrent Tumors				
	Recurrent cell line #1	Recurrent cell line #2	Rec.1	Rec.2	Rec.3	Rec.8	Rec.10	Prim.1	Prim.4	Prim.5	Rec.6	Rec.9	Rec.5	Rec.4	Rec.11
PIK3CA	E542K	None	None	None	None	None	None	None	None	None	None	None	None	None	None
TP53	None	None	None	None	None	None	None	None	None	None	None	None	None	None	None
KMT2C	None	None	None	None	None	None	None	None	None	None	None	None	T3734A	None	None
GATA3	None	None	None	None	None	None	None	None	None	None	None	None	None	None	None
CDH1	None	None	None	None	G581D	None	None	R547S	None	None	None	None	None	None	None
MAP3K1	None	None	None	None	None	None	None	None	None	None	None	None	None	None	None
KMT2D	None	Indel H3611	Indel H3611	None	None	None	S885L	F5236L, S885L	None	None	Indel H3611	None	None	None	S885L
PDE4DIP	None	None	None	None	None	None	None	K228N	None	G2200D	None	None	None	None	None
TBX3	None	Y251H	E306D	None	None	None	None	None	None	None	A694T, E306D	None	None	None	None
NOTCH1	None	None	None	None	None	None	None	None	None	None	None	None	None	None	None
ARID1A	None	None	None	None	None	None	None	None	None	None	None	None	None	None	None
CBFB	None	None	None	None	None	None	None	None	None	R198C	None	None	None	None	None
NCOR2	None	None	None	None	None	None	None	None	None	None	None	None	None	None	None
AKT1	None	None	None	None	None	None	None	None	None	None	None	None	None	None	None
PTEN	None	None	None	None	None	None	None	None	None	None	None	None	None	None	None
ATR	None	None	None	None	None	V2296M	None	None	None	None	None	None	None	None	None
NF1	None	None	I558S	None	I558S	No	None	None	None	G1135D, E413D	None	None	I558S	I558S	I558S
NCOR1	None	None	None	None	Indel Q208, S51N	No	None	V1247L	None	None	None	E180D	None	None	None
MYH9	None	None	None	None	None	None	None	None	None	None	None	None	None	None	None
ROS1	None	None	None	None	None	None	None	None	None	None	None	None	None	None	None

Supplementary Table 2. Tumors and tumor cell lines used in this study.

Tumor or cell line description	Source	Figures
Primary tumor cell line #1 (from donor tumor #1)	Autochthonous MTB;TAN primary tumor	Figure 1, Figure 2, Figure 5<br><br>Supplemental Figure 1, 2, 4
Barcoded orthotopic primary tumors #1 - 6	Orthotopic primary tumors arising from injection of barcoded primary tumor cell line #1	Figure 2<br><br>Supplemental Figure 3, 5, 6, 7, 8
Late orthotopic primary tumors #1-6	Orthotopic primary tumors arising from injection of barcoded primary tumor cell line #1. Tumors were grown until maximum allowed tumor volume	Supplemental Figure 6
Barcoded orthotopic recurrent tumors #1 - 12	Orthotopic recurrent tumors arising from injection of barcoded primary tumor cell line #1	Figure 3, Figure 4<br><br>Supplemental Figure 7, 8
1668 and 1669	Cell lines generated from orthotopic recurrent tumors arising from a separate injection of barcoded primary tumor cell line #1.	Figure 4<br><br>Supplemental Figure 10
Primary tumor cell line #2 (from donor tumor #2)	Autochthonous MTB;TAN primary tumor	Figure 5<br><br>Supplemental Figure 4, 9
Barcoded orthotopic primary tumors #1 - 4	Orthotopic primary tumors arising from injection of barcoded primary tumor cell line #2	Figure 5<br><br>Supplemental Figure 9, 10
Barcoded orthotopic recurrent tumors #1 - 8	Orthotopic recurrent tumors arising from injection of barcoded primary tumor cell line #2	Figure 5<br><br>Supplemental Figure 9, 10
Recurrent tumor cell line #1	Autochthonous MTB;TAN recurrent tumor without Met amplification	Figure 5<br><br>Supplemental Figure 4, 8
Recurrent tumor cell line #2	Autochthonous MTB;TAN recurrent tumor without Met amplification	Figure 5<br><br>Supplemental Figure 8
Recurrent tumor cell line #3	Autochthonous MTB;TAN recurrent tumor with Met amplification	Supplemental Figure 4, 8

Supplementary Table 3. Primer sequences for barcode sequencing.

Primer name	Sequence
**Step1_F**	5’ GCCTCCCTCGCGCCATCAGAGATAGAGGTTCAGAGTTCTACAGTCCGAA 3’
**Step1_R**	5’ GTGACTGGAGTTCAGACGTGTGCTCTTCCGATCT**NNNN**TCAAGCAGAAGACGGCATACGAAGACA3’
**PCR Primer, Index 1**	CAAGCAGAAGACGGCATACGAGAT**CGTGAT**GTGACTGGAGTTCAGACGTGTGCTC
**PCR Primer, Index 2**	CAAGCAGAAGACGGCATACGAGAT**ACATCG**GTGACTGGAGTTCAGACGTGTGCTC
**PCR Primer, Index 3**	CAAGCAGAAGACGGCATACGAGAT**GCCTAA**GTGACTGGAGTTCAGACGTGTGCTC
**PCR Primer, Index 4**	CAAGCAGAAGACGGCATACGAGAT**TGGTCA**GTGACTGGAGTTCAGACGTGTGCTC
**PCR Primer, Index 5**	CAAGCAGAAGACGGCATACGAGAT**CACTGT**GTGACTGGAGTTCAGACGTGTGCTC
**PCR Primer, Index 6**	CAAGCAGAAGACGGCATACGAGAT**ATTGGC**GTGACTGGAGTTCAGACGTGTGCTC
**PCR Primer, Index 7**	CAAGCAGAAGACGGCATACGAGAT**GATCTG**GTGACTGGAGTTCAGACGTGTGCTC
**PCR Primer, Index 8**	CAAGCAGAAGACGGCATACGAGAT**TCAAGT**GTGACTGGAGTTCAGACGTGTGCTC
**PCR Primer, Index 9**	CAAGCAGAAGACGGCATACGAGAT**CTGATC**GTGACTGGAGTTCAGACGTGTGCTC
**PCR Primer, Index 10**	CAAGCAGAAGACGGCATACGAGAT**AAGCTA**GTGACTGGAGTTCAGACGTGTGCTC
**PCR Primer, Index 11**	CAAGCAGAAGACGGCATACGAGAT**GTAGCC**GTGACTGGAGTTCAGACGTGTGCTC
**PCR Primer, Index 12**	CAAGCAGAAGACGGCATACGAGAT**TACAAG**GTGACTGGAGTTCAGACGTGTGCTC
**PCR Primer, Index 13**	CAAGCAGAAGACGGCATACGAGAT**TATGGA**GTGACTGGAGTTCAGACGTGTGCTC
**PCR Primer, Index 14**	CAAGCAGAAGACGGCATACGAGAT**TAGTAC**GTGACTGGAGTTCAGACGTGTGCTC
**PCR Primer, Index 15**	CAAGCAGAAGACGGCATACGAGAT**TACTGT**GTGACTGGAGTTCAGACGTGTGCTC
**PCR Primer, Index 16**	CAAGCAGAAGACGGCATACGAGAT**CATGAG**GTGACTGGAGTTCAGACGTGTGCTC
**PCR Primer, Index 17**	CAAGCAGAAGACGGCATACGAGAT**TATCGT**GTGACTGGAGTTCAGACGTGTGCTC
**PCR Primer, Index 18**	CAAGCAGAAGACGGCATACGAGAT**TCTGCA**GTGACTGGAGTTCAGACGTGTGCTC
**PCR Primer, Index 19**	CAAGCAGAAGACGGCATACGAGAT**TATGAA**GTGACTGGAGTTCAGACGTGTGCTC
**PCR Primer, Index 20**	CAAGCAGAAGACGGCATACGAGAT**ACAGCA**GTGACTGGAGTTCAGACGTGTGCTC
**PCR Primer, Index 21**	CAAGCAGAAGACGGCATACGAGAT**GTGATA**GTGACTGGAGTTCAGACGTGTGCTC
**PCR Primer, Index 22**	CAAGCAGAAGACGGCATACGAGAT**TATCCA**GTGACTGGAGTTCAGACGTGTGCTC
**PCR Primer, Index 23**	CAAGCAGAAGACGGCATACGAGAT**AATGCG**GTGACTGGAGTTCAGACGTGTGCTC
**PCR Primer, Index 24**	CAAGCAGAAGACGGCATACGAGAT**TAAGGC**GTGACTGGAGTTCAGACGTGTGCTC
**ClonalBarcodeAdaptor_1**	AATGATACGGCGACCACCGAGATCTACACGCCTCCCTCGCGCCATCAGAGATAG
**Clonal Barcodes R1**	5’ CCTCGCGCCATCAGAGATAGAGGTTCAGAGTTCTACAGTCCGAA 3’

Supplementary Table 4: Summary of alignment against mouse reference genome

	4598-S1 Pri	4598-S2 Pri	4598-S3 Pri	4598-S4 Pri	4598-S5 Rec	4598-S6 Rec
Number of input reads |	42659074	29540441	38727376	24948617	30646176	35420289
Average input read length |	51	51	51	51	51	51
UNIQUE READS:						
Uniquely mapped reads number |	30538542	19896016	28791765	14976186	23589193	24161181
Uniquely mapped reads % |	71.59%	67.35%	74.34%	60.03%	76.97%	68.21%
Average mapped length |	50.67	50.68	50.74	50.63	50.79	50.80
Number of splices: Total |	2669425	1590364	2995345	1046825	2864610	3157165
Number of splices: Annotated (sjdb) |	2656793	1583032	2983434	1040207	2851796	3145975
Number of splices: GT/AG |	2609461	1549460	2938611	1019339	2795545	3058397
Number of splices: GC/AG |	46252	33285	43235	20708	54320	85295
Number of splices: AT/AC |	2541	1480	2701	942	2711	3031
Number of splices: Non-canonical |	11171	6139	10798	5836	12034	10442
Mismatch rate per base, % |	0.45%	0.50%	0.33%	0.67%	0.27%	0.26%
Deletion rate per base |	0.01%	0.01%	0.01%	0.01%	0.01%	0.01%
Deletion average length |	1.78	1.68	1.73	2.06	1.95	1.73
Insertion rate per base |	0.01%	0.01%	0.01%	0.01%	0.01%	0.00%
Insertion average length |	1.42	1.37	1.38	1.21	1.37	1.33
MULTI-MAPPING READS:						
Number of reads mapped to multiple loci |	9750199	7838261	8604523	7814341	6261030	10391146
% of reads mapped to multiple loci |	22.86%	26.53%	22.22%	31.32%	20.43%	29.34%
Number of reads mapped to too many loci |	338377	206081	333295	152053	252132	380601
% of reads mapped to too many loci |	0.79%	0.70%	0.86%	0.61%	0.82%	1.07%
UNMAPPED READS:						
% of reads unmapped: too many mismatches |	0.00%	0.00%	0.00%	0.00%	0.00%	0.00%
% of reads unmapped: too short |	3.97%	4.55%	1.81%	7.52%	1.16%	0.68%
% of reads unmapped: other |	0.80%	0.86%	0.77%	0.53%	0.61%	0.69%
CHIMERIC READS:						
Number of chimeric reads |	0	0	0	0	0	0
% of chimeric reads |	0.00%	0.00%	0.00%	0.00%	0.00%	0.00%

	4598-S7 Rec	4598-S8 Rec	4598-S9 Rec	4598-S10 Rec	4598-S11 Rec	4598-S12 Rec
Number of input reads |	34255088	26886332	29892965	33796875	29075545	28160651
Average input read length |	51	51	51	51	51	51
UNIQUE READS:						
Uniquely mapped reads number |	22261233	12180521	15666989	13706947	13171114	18973177
Uniquely mapped reads % |	64.99%	45.30%	52.41%	40.56%	45.30%	67.37%
Average mapped length |	50.81	50.13	50.35	49.74	50.22	50.71
Number of splices: Total |	3246886	301737	66276	364136	723084	2249561
Number of splices: Annotated (sjdb) |	3238641	298605	64189	359727	718566	2243333
Number of splices: GT/AG |	3142707	295050	63208	339200	695068	2208575
Number of splices: GC/AG |	92898	4032	1351	20883	23644	33334
Number of splices: AT/AC |	3306	225	64	338	674	1952
Number of splices: Non-canonical |	7975	2430	1653	3715	3698	5700
Mismatch rate per base, % |	0.26%	1.16%	1.30%	1.55%	0.97%	0.41%
Deletion rate per base |	0.01%	0.01%	0.01%	0.02%	0.02%	0.01%
Deletion average length |	1.78	1.23	1.11	1.53	1.39	1.80
Insertion rate per base |	0.00%	0.01%	0.00%	0.01%	0.01%	0.01%
Insertion average length |	1.34	1.29	1.15	1.16	1.19	1.30
MULTI-MAPPING READS:						
Number of reads mapped to multiple loci |	11325955	11897339	11997887	11617847	10731316	7940197
% of reads mapped to multiple loci |	33.06%	44.25%	40.14%	34.38%	36.91%	28.20%
Number of reads mapped to too many loci |	347612	273878	191877	175775	216190	207089
% of reads mapped to too many loci |	1.01%	1.02%	0.64%	0.52%	0.74%	0.74%
UNMAPPED READS:						
% of reads unmapped: too many mismatches |	0.00%	0.00%	0.00%	0.00%	0.00%	0.00%
% of reads unmapped: too short |	0.62%	9.22%	6.57%	24.43%	16.62%	3.00%
% of reads unmapped: other |	0.32%	0.21%	0.24%	0.12%	0.43%	0.70%
CHIMERIC READS:						
Number of chimeric reads |	0	0	0	0	0	0
% of chimeric reads |	0.00%	0.00%	0.00%	0.00%	0.00%	0.00%
