# Supplementary material for: Adaptation and selection shape clonal evolution of tumors during residual disease and recurrence
Source: Nat Commun. 2020 Oct 6;11:5017. doi: 10.1038/s41467-020-18730-z (PMC7539014; doi:10.1038/s41467-020-18730-z)
Supplement: Supplementary file 3 — Description of Additional Supplementary Files [file 41467_2020_18730_MOESM3_ESM.pdf]

Description of Additional Supplementary Files

File Name: Supplementary Data 1
Description: Top 50 genes differentially expressed in each cluster from pairwise comparison between primary cell line #1 and recurrent cell line #3.

File Name: Supplementary Data 2
Description: Top 50 genes differentially expressed in each cluster from pairwise comparison between primary cell line #1 and recurrent cell line #1.

File Name: Supplementary Data 3
Description: Gene sets enriched in cells with Her2 expression (+dox).

File Name: Supplementary Data 4
Description: Gene sets enriched in cells following Her2 downregulation (-dox).

File Name: Supplementary Data 5
Description: DESeq2 results showing differentially expressed genes in recurrent as compared to primary tumors.

File Name: Supplementary Data 6
Description: DESeq2 results showing differentially expressed genes between Met amplified and non-amplified recurrent tumors.
