# Supplementary material for: Adaptation and selection shape clonal evolution of tumors during residual disease and recurrence
Source: Nat Commun. 2020 Oct 6;11:5017. doi: 10.1038/s41467-020-18730-z (PMC7539014; doi:10.1038/s41467-020-18730-z)
Supplement: Supplementary file 10 — Reporting Summary [file 41467_2020_18730_MOESM10_ESM.pdf]

nature research

Corresponding author(s): James V. Alvarez

Last updated by author(s): Aug 3, 2020

Reporting Summary

Nature Research wishes to improve the reproducibility of the work that we publish. This form provides structure for consistency and transparency in reporting. For further information on Nature Research policies, see our Editorial Policies and the Editorial Policy Checklist.

Statistics

For all statistical analyses, confirm that the following items are present in the figure legend, table legend, main text, or Methods section.

n/a	Confirmed	
☐	☒	The exact sample size (*n*) for each experimental group/condition, given as a discrete number and unit of measurement
☐	☒	A statement on whether measurements were taken from distinct samples or whether the same sample was measured repeatedly
☐	☒	The statistical test(s) used AND whether they are one- or two-sided<br>*Only common tests should be described solely by name; describe more complex techniques in the Methods section.*
☐	☒	A description of all covariates tested
☐	☒	A description of any assumptions or corrections, such as tests of normality and adjustment for multiple comparisons
☐	☒	A full description of the statistical parameters including central tendency (e.g. means) or other basic estimates (e.g. regression coefficient) AND variation (e.g. standard deviation) or associated estimates of uncertainty (e.g. confidence intervals)
☐	☒	For null hypothesis testing, the test statistic (e.g. *F*, *t*, *r*) with confidence intervals, effect sizes, degrees of freedom and *P* value noted<br>*Give P values as exact values whenever suitable.*
☒	☐	For Bayesian analysis, information on the choice of priors and Markov chain Monte Carlo settings
☒	☐	For hierarchical and complex designs, identification of the appropriate level for tests and full reporting of outcomes
☐	☒	Estimates of effect sizes (e.g. Cohen's *d*, Pearson's *r*), indicating how they were calculated

Our web collection on statistics for biologists contains articles on many of the points above.

Software and code

Policy information about availability of computer code

Data collection	Biorad CFX Manager Version 3.1 was used to acquire qPCR data.<br>Zeiss AxioImager Z2 microscope with Zeiss ZEN 2 software Version 3.2 was used for microscopy.<br>Gen5 2.06 software was used to collect cell titer glo data.<br>Bioluminescence images were acquired using IVIS Lumina III bioluminescence/fluorescence imager (PerkinElmer) and data were analyzed using LivingImage 4.7.3 (PerkinElmer).
Data analysis	Graphpad Prism 8 was used for statistical analyses of qPCR and cell viability. For barcode sequencing, statistical analyses were mainly scripted using the R statistical environment[R] (version 3.4.4 and 3.6.1) along with its extension packages from the comprehensive R archive network (CRAN; https://cran.r-project.org/) and the Bioconductor project[BIOC]. All analysis were performed and documented using git (https://git-scm.com) for source code management in gitlab (https://gitlab.oit.duke.edu/). SIngle-cell RNA-sequencing data were analyzed using 10X Genomic's Cell Ranger software (version 3.1.0). Secondary statistical analysis of scRNA-seq data was performed using the R package Seurat (version 3.2.0). Gene differential expression was performed within the framework of a negative binomial model using R (v3.4.4) (R Core Team, R: A Language and Environment for Statistical Computing. 2016: Vienna, Austria.) and its extension package DESeq2 (v1.18.1). Code for barcode mapping and data analysis is available at [https://gitlab.oit.duke.edu/dcibioinformatics/pubs/alvarez-barcode-paper].

For manuscripts utilizing custom algorithms or software that are central to the research but not yet described in published literature, software must be made available to editors and reviewers. We strongly encourage code deposition in a community repository (e.g. GitHub). See the Nature Research guidelines for submitting code & software for further information.

Data

Policy information about availability of data

All manuscripts must include a data availability statement. This statement should provide the following information, where applicable:

- Accession codes, unique identifiers, or web links for publicly available datasets
- A list of figures that have associated raw data
- A description of any restrictions on data availability

All sequencing data, including barcode sequencing, RNA sequencing, and whole-exome sequencing are available at Sequence Read Archive (https://www.ncbi.nlm.nih.gov/Traces/study/?acc=PRJNA509416).

Field-specific reporting

Please select the one below that is the best fit for your research. If you are not sure, read the appropriate sections before making your selection.

☒ Life sciences ☐ Behavioural & social sciences ☐ Ecological, evolutionary & environmental sciences

For a reference copy of the document with all sections, see nature.com/documents/nr-reporting-summary-flat.pdf

Life sciences study design

All studies must disclose on these points even when the disclosure is negative.

Sample size	We did not calculate sample sizes prior to performing in vitro experiments, but sample sizes were chosen based upon previously published papers, including:<br>• Walens A, DiMarco, AV, Lupo, R, Kroger, BR, Damrauer, JS & Alvarez, JV. CCL5 promotes breast cancer recurrence through macrophage recruitment in residual tumors. Elife 8, (2019).<br>• Fox DB, Garcia, NMG, McKinney, BJ, Lupo, R, Noteware, LC, Newcomb, R, Liu, J, Locasale, JW, Hirschey, MD & Alvarez, JV. NRF2 activation promotes the recurrence of dormant tumour cells through regulation of redox and nucleotide metabolism. Nature Metabolism 2, 318-334, (2020).<br>• Mabe NW, Fox, DB, Lupo, R, Decker, AE, Phelps, SN, Thompson, JW & Alvarez, JV. Epigenetic silencing of tumor suppressor Par-4 promotes chemoresistance in recurrent breast cancer. J Clin Invest 128, 4413-4428, (2018).<br><br>For barcode sequencing of tumors in vivo, the sample size (n=6 for primary and residual tumors; n=12 for recurrent tumors) was sufficient to capture within group variability, as evidenced by the high correlation in barcode composition within groups. For in vivo drug studies (e.g. Figure S8B), we determined the minimum number of mice needed to detect a statistically significant difference (p<0.05) with a power of 0.8 with an assumed standard deviation of 30%. All n values are reported.
Data exclusions	After optimizing experimental conditions for assays, no data were excluded.
Replication	All in vitro experiments, including western blotting, qPCR, and CellTiterGlo experiments were successfully repeated 2-3 times, and all attempts to replicate experiments were successful. Mouse experiments included between n=6 (primary, late primary, early residual, late residual tumors) or n=12 (recurrent tumors) independent mice. In addition, primary and recurrent time-points were repeated with an independent primary donor tumor.
Randomization	For barcode experiments, recipient mice were littermates and were randomly assigned to cohorts. For tumor growth experiments, mice with tumors were randomly assigned to vehicle of drug treatment cohorts. Experiments were not randomized. For in vitro experiments, cells were plated at the same time and wells were randomly assigned to treatments.
Blinding	Microscopy images of immunofluorescence staining were collected in a blinded manner. Other in vitro experiments were not blinded since data collection was either automated (qPCR, CellTiterGlo) or the investigator needed to know the sample identity to execute the experiment (western blotting). For in vivo experiments, investigators were blinded to the experimental cohorts during tumor measurements.

Reporting for specific materials, systems and methods

We require information from authors about some types of materials, experimental systems and methods used in many studies. Here, indicate whether each material, system or method listed is relevant to your study. If you are not sure if a list item applies to your research, read the appropriate section before selecting a response.

Materials & experimental systems

n/a	Involved in the study
☐	☒ Antibodies
☐	☒ Eukaryotic cell lines
☒	☐ Palaeontology and archaeology
☐	☒ Animals and other organisms
☒	☐ Human research participants
☒	☐ Clinical data
☒	☐ Dual use research of concern

Methods

n/a	Involved in the study
☒	☐ ChIP-seq
☒	☐ Flow cytometry
☒	☐ MRI-based neuroimaging

Antibodies

Antibodies used	phospho-Stat3, Cell Signaling #9145. Stat3, Cell Signaling #4904. E-cadherin, Cell Signaling #3195. alpha-Tubulin, Cell Signaling #3873.
Validation	Antibodies were chosen based on previous publications using the same antibodies and manufacturer's verification, which includes a reduction in signal with siRNA-mediated knockdown for Stat3, increased signal with cytokine stimulation for pStat3, and appropriate membrane localization for E-cadherin.

Eukaryotic cell lines

Policy information about cell lines

Cell line source(s)	Primary cell cultures (not immortalized cell lines) were derived from primary or recurrent mammary tumors arising in MMTV-rtTA;TetO-Her2/neu mice.
Authentication	Cell lines were not authenticated since they are unique to this study and there is no standard against which to authenticate them.
Mycoplasma contamination	All cell lines were tested for mycoplasma contamination by the Duke Cell Culture Facility and tested negative.
Commonly misidentified lines (See ICLAC register)	No commonly misidentified cell lines were used.

Animals and other organisms

Policy information about studies involving animals; ARRIVE guidelines recommended for reporting animal research

Laboratory animals	This study used immunocompromised athymic nude (Foxn1 nu) female mice at 6-weeks of age for all tumor cell injections.
Wild animals	This study did not involve wild animals
Field-collected samples	This study did not involve samples collected from the field.
Ethics oversight	All experiments were approved by Duke IACUC (Approval #A199-17-08).

Note that full information on the approval of the study protocol must also be provided in the manuscript.
