# Supplementary figures and images for: Adaptation and selection shape clonal evolution of tumors during residual disease and recurrence
Source: Nat Commun. 2020 Oct 6;11:5017. doi: 10.1038/s41467-020-18730-z (PMC7539014; doi:10.1038/s41467-020-18730-z)

## Slide 1
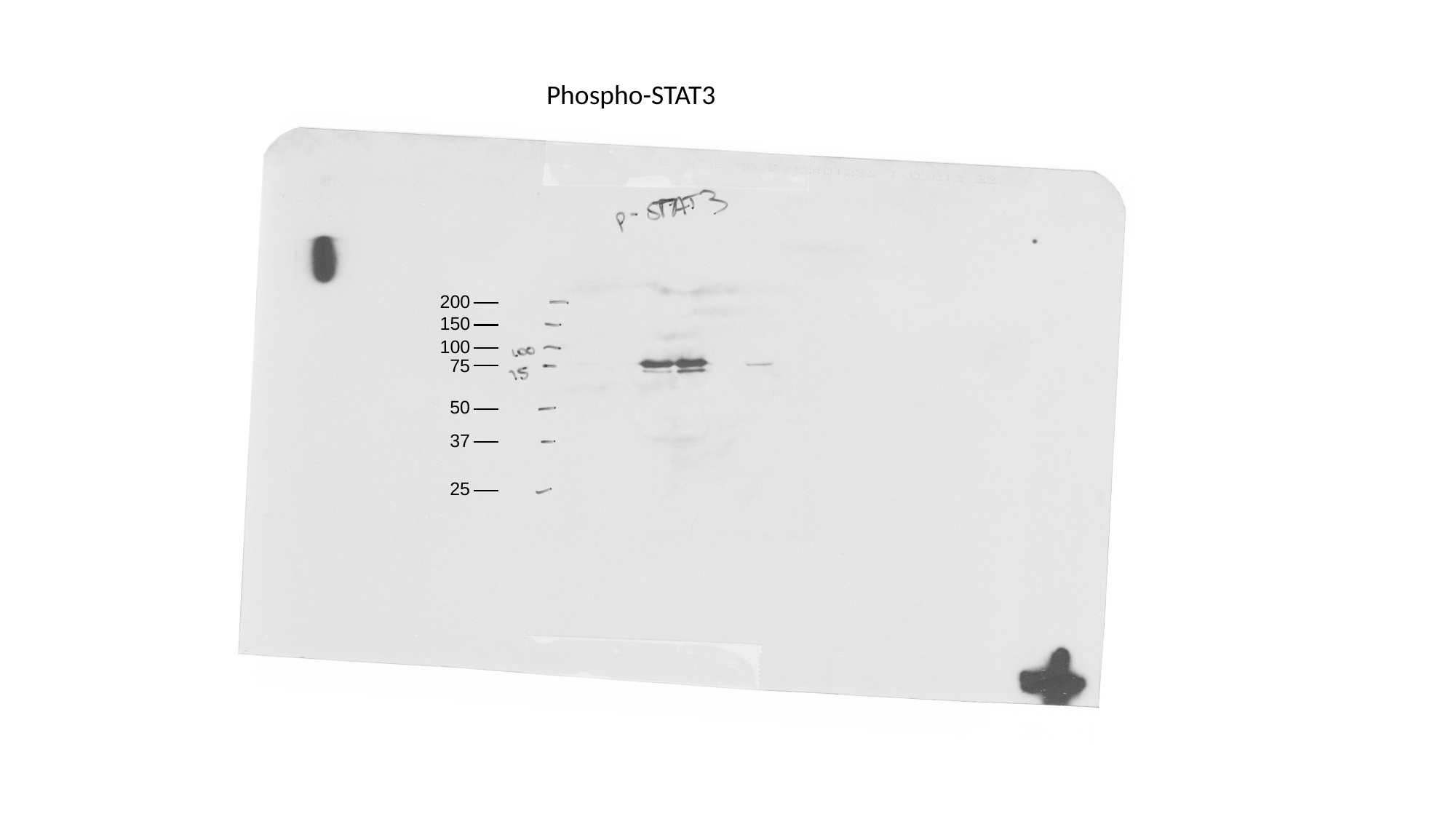

Phospho-STAT3
200
150
100
75
50
37
25

## Slide 2
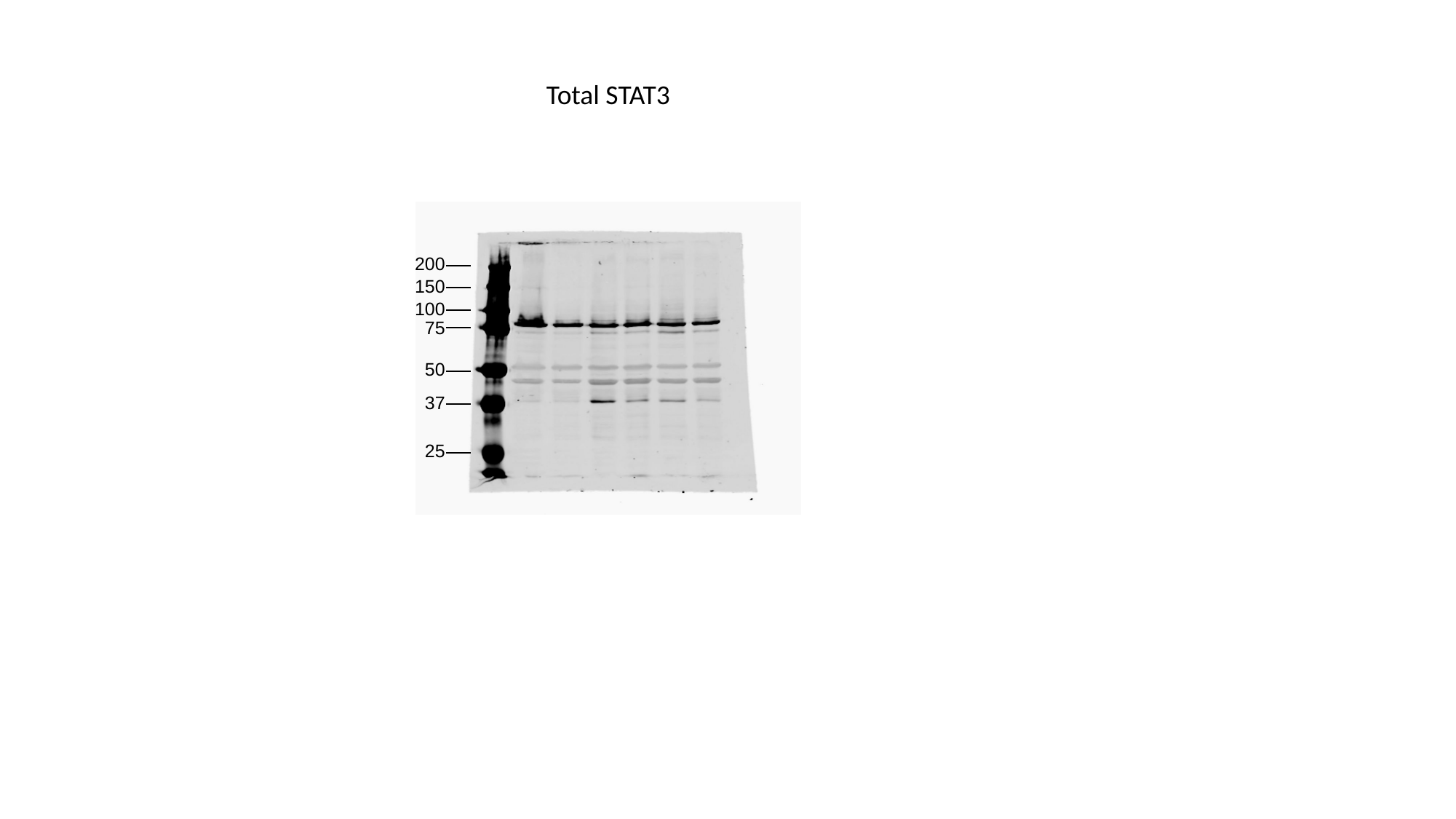

Total STAT3
200
150
100
75
50
37
25

## Slide 3
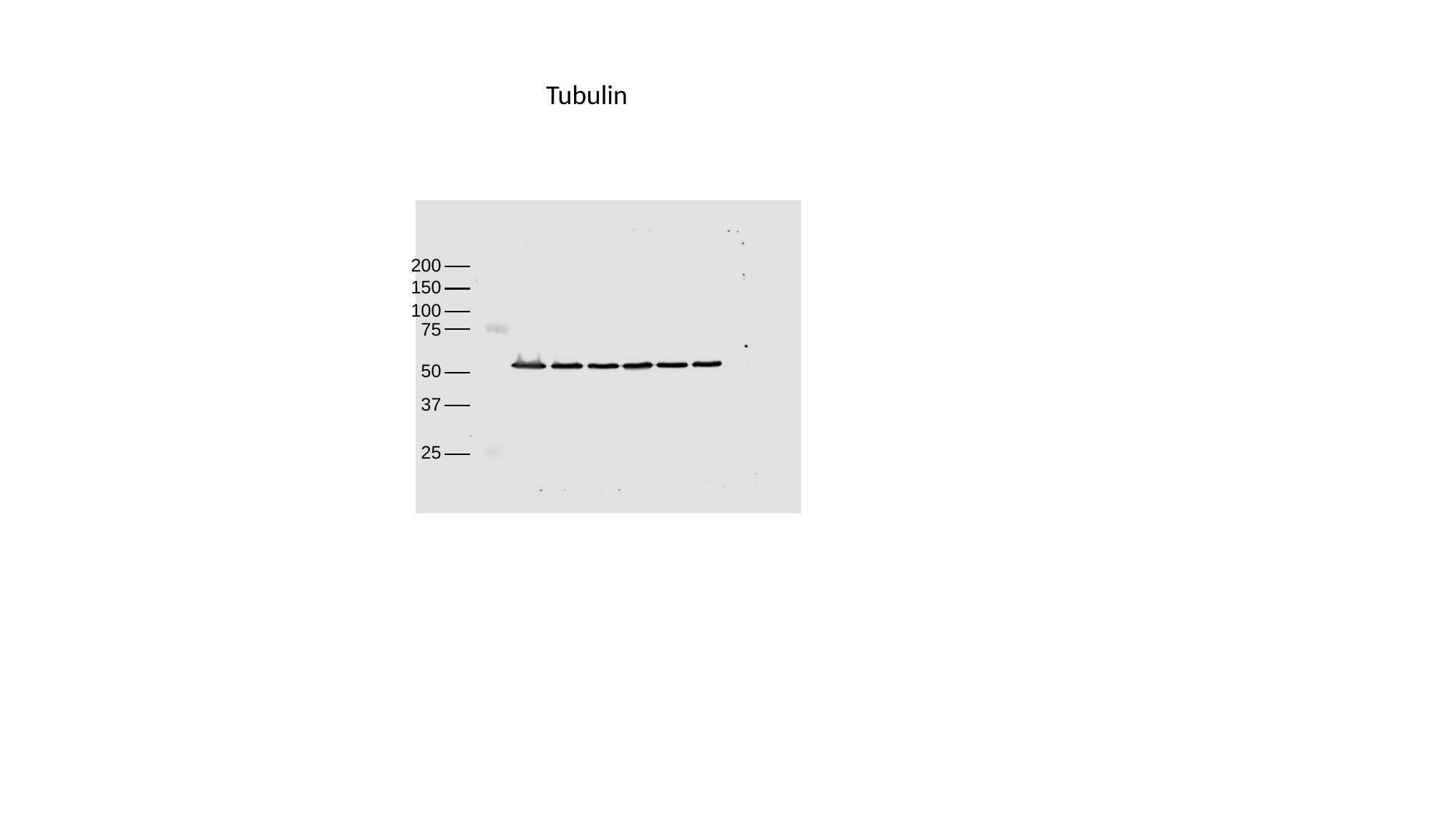

Tubulin
200
150
100
75
50
37
25

Supplement: Supplementary file 11 — Source Data [file 41467_2020_18730_MOESM11_ESM.pptx]
